# Natural Compounds and Histone Deacetylase Inhibitors: A Combined Approach Against mCRPC Cells

**DOI:** 10.3390/biomedicines13020296

**Published:** 2025-01-25

**Authors:** Janiah Alimudin, Zeynep Betts, Asuman Deveci Ozkan

**Affiliations:** 1 Department of Biology, Institute of Science, Kocaeli University, Kocaeli 41001, Türkiye; jenayiah18@gmail.com; 2 Manchester Institute of Biotechnology, Faculty of Science and Engineering, University of Manchester, Menchester M1 7DN, UK; duman.zeynep@gmail.com; 3 Department of Biology, Faculty of Science and Art, Kocaeli University, Kocaeli 41001, Türkiye; 4 Department of Medical Biology, Faculty of Medicine, Sakarya University, Sakarya 54290, Türkiye

**Keywords:** prostate cancer, rutin, sodium butyrate, combination effect, apoptosis

## Abstract

**Background**: Sodium butyrate (NaBu), a short-chain fatty acid, modulates global gene expression through histone deacetylase (HDAC) inhibition, suppressing proliferation and inducing apoptosis in various cancers. Rutin (RUT), a polyphenolic flavonoid found in many plants, exhibits notable anticancer properties. Combining chemotherapeutic agents with natural polyphenols represents a promising strategy for cancer therapy. This study aims to evaluate, for the first time, the potential effects of NaBu and RUT combination therapy on metastatic castration-resistant prostate cancer (mCRPC) cells. **Methods**: PC-3 cells were treated with varying concentrations of NaBu, RUT, and their combinations. Cell viability was assessed using the WST-1 assay. Based on combination index values, selected treatments were further analyzed for apoptosis (Annexin V assay), intracellular reactive oxygen species (ROS) production, mRNA expression levels, and changes in cell and nuclear morphology. **Results**: The combined treatment of NaBu and RUT significantly reduced cell viability compared to individual treatments. Enhanced apoptotic induction and elevated ROS levels were observed in combination-treated cells, alongside notable changes in cellular and nuclear morphology and mRNA expression levels. **Conclusions**: NaBu and RUT combination therapy exhibits a synergistic anticancer effect in mCRPC cells by inhibiting cell viability, inducing apoptosis, and increasing ROS production. These findings suggest a promising therapeutic approach that warrants further investigation to elucidate the underlying molecular mechanisms and assess its potential in preclinical and clinical settings.

## 1. Introduction

Prostate cancer (PCa), currently the second leading cause of cancer-related mortality in males within industrialized nations, has seen an increase in incidence over recent years [1]. Metastatic castration-resistant prostate cancer (mCRPC) is characterized by disease progression despite testosterone deprivation or surgical castration. This progression is driven by the persistence of androgen receptors (AR) and the activation of the androgen axis [2,3]. The complexity of cellular mechanisms involved in PCa development poses significant challenges for treatment [4]. To mitigate the side effects of current PCa therapies, alternative strategies are being explored, including the use of various bioactive compounds either as standalone treatments or in combination with chemotherapeutic agents [5,6].

Histone deacetylase (HDAC) enzymes, which regulate the acetylation and deacetylation of histone proteins, have emerged as promising therapeutic targets [7]. Sodium butyrate (NaBu), a short-chain fatty acid, acts as an HDAC inhibitor, influencing chromatin remodeling, altering global gene expression, reducing cell proliferation, and inducing apoptosis at low concentrations. It has shown potential as an anticancer agent in various cancer cell types [8,9]. Rutin (RUT), a polyphenolic antioxidant abundant in foods such as green tea and apples, has demonstrated a range of biological effects, including anti-carcinogenic, neuroprotective, anti-proliferative, anti-inflammatory, and anti-oxidative properties by inhibiting lipid peroxidation [10]. Additionally, RUT has been shown to induce apoptosis in various cancer cell lines [11,12].

The synergistic interaction between natural polyphenols and chemotherapeutic agents can enhance cancer treatment efficacy by inhibiting cell proliferation and promoting apoptosis [13]. Moreover, combination therapies involving herbal compounds have been reported to reduce drug resistance and mitigate chemotherapy-related complications [12]. Clinical studies have demonstrated that flavonoids administered alongside chemotherapeutic agents improve tumor suppression and patient survival outcomes [14].

The combination of natural polyphenols with chemotherapeutic drugs holds great promise as a strategy for cancer treatment. Natural agents, may reduce the likelihood of the severe side effects commonly associated with synthetic chemotherapeutic agents, thereby offering a more tolerable treatment option for patients. By focusing on the synergistic potential of sodium butyrate and rutin, we can develop a more effective, multi-pronged approach to cancer treatment that enhances therapeutic outcomes, improves patient tolerance, and offers the potential for use in combination with conventional chemotherapy regimens. This dual-target strategy may provide significant advantages in overcoming resistance mechanisms, reducing side effects, and improving overall treatment efficacy. Therefore, this study aims to investigate, for the first time, the combined therapeutic effects of RUT and NaBu on mCRPC PC-3 cells. To achieve this, PC-3 cells were treated with RUT, NaBu, and their combination, and various parameters were assessed, including Annexin V levels, intracellular reactive oxygen species (ROS), mRNA and protein expression levels, as well as cell and nuclear morphology.

## 2. Materials and Methods

### 2.1. Cell Culture

In this study, the PC-3 cell line was used as a model for mCRPC cells, while human umbilical vein endothelial cells (HUVEC) were employed to evaluate the cytotoxicity of the combined treatments specifically. Both cell lines were commercially obtained from the American Type Culture Collection (ATCC, Manassas, VA, USA). PC-3 cells were cultured in RPMI-1640 medium (Sigma Aldrich, St. Louis, MO, USA), while HUVEC cells were grown in Dulbecco’s Modified Eagle’s Medium (DMEM). The culture media for both of the cell lines were supplemented with 10% heat-inactivated fetal bovine serum (FBS) and 1% penicillin–streptomycin solution (Sigma Aldrich, St. Louis, MO, USA). The cells were maintained in a humidified incubator (Thermo Fisher Scientific, Waltham, MA, USA) set to 37 °C with 5% CO_2_.

### 2.2. Cell Viability Assay

In this study, NaBu and RUT will be administered to cells both separately and in combination. The use of these two compounds, derived from natural sources, offers a versatile approach to address key challenges in cancer treatment, such as inhibiting cancer cell proliferation and promoting apoptosis. When combined, NaBu and RUT may exert synergistic effects on cancer cells by targeting multiple cellular pathways involved in tumor progression [13]. NaBu’s epigenetic modulation and ability to induce cell cycle arrest complement RUT’s ability to disrupt key survival and proliferation signals, amplifying their individual anticancer properties [7,10]. Stock solutions of RUT (Tokyo Chemical Industry, Tokyo, Japan) and NaBu (Sigma Aldrich, USA) were prepared according to the manufacturers’ instructions. PC-3 and HUVEC cells were treated with varying concentrations of RUT (0–1000 µM) and NaBu (0–10 mM), either individually or in combination, to assess cell viability using the WST-1 assay. For the assay, 96-well plates were seeded with approximately 2 × 10^4^ cells per well of PC-3 and HUVEC cells. The cells were exposed to RUT, NaBu, or their combinations at different concentrations for 24 and 48 h. Following the incubation period, WST-1 reagent (Biovision, CA, USA) was added to each well, and the plates were incubated at 37 °C for 30 min. Cell viability was then measured using a microplate reader at 450 nm (Chromate, Shijiazhuang, China). Each experiment was performed in triplicate for both cell lines. The most effective combination concentrations of RUT and NaBu, along with the optimal exposure time, were determined by calculating the combination index (CI) and drug reduction index (DRI) values. HUVEC cells were used only in the viability assay.

### 2.3. Enzyme-Linked Immunosorbent Assay (ELISA)

Annexin A5 (ANXA5) is a 36 kDa protein that interacts with phospholipids in a calcium-dependent manner, with phosphatidylserine (PS) being its preferred binding partner. ANXA5 serves as a valuable tool for identifying apoptotic cells due to its ability to bind PS [15]. To evaluate the apoptotic effects of RUT and NaBu, both individually and in combination, an ELISA assay was conducted to quantify ANXA5 levels in only PC-3 cells. PC-3 cells (5 × 10^4^ cells/well) were seeded into a 96-well plate and treated with RUT (500 and 750 µM), NaBu (1 and 2.5 mM), or their combinations for 48 h. Additionally, a control group was established, cultured under the same conditions but without the addition of RUT, NaBu, or their combination. After the incubation period, cell culture supernatants were collected from each treatment group and analyzed using the Human Annexin V ELISA Kit (Abexxa, Cambridge, UK) following the manufacturer’s instructions. The results obtained were compared with this control group, which received no treatment, and were analyzed statistically.

### 2.4. ROS Measurement

To assess the impact of RUT and NaBu, both individually and in combination, on intracellular ROS levels, a cellular ROS assay was conducted. PC-3 cells (4 × 10⁶ cells/well) were seeded into a 96-well plate and treated with RUT (500 and 750 µM), NaBu (1 and 2.5 mM), or their combinations for 48 h. Additionally, a control group was established, cultured under the same conditions but without the addition of RUT, NaBu, or their combination. Following the incubation period, intracellular ROS levels were measured using the DCFDA/H2DCFDA-Cellular ROS Assay Kit (Abcam, Cambridge, UK) in accordance with the manufacturer’s instructions. The results obtained were compared with this control group, which received no treatment, and were analyzed statistically.

### 2.5. Cell and Nuclear Morphology Observation

To assess changes in cell and nuclear morphology following the treatment with RUT and NaBu, both individually and in combination, Acridine Orange (AO) and 4′,6-Diamidino-2-phenylindole dihydrochloride (DAPI) staining was performed. PC-3 cells (4 × 10⁵ cells/well) were cultured in a 6-well plate with slides and treated with the most effective combined concentrations of RUT (500 and 750 µM) and NaBu (1 and 2.5 mM) for 48 h. Additionally, a control group was established, cultured under the same conditions but without the addition of RUT, NaBu, or their combination. After the incubation period, the cells were fixed with 4% paraformaldehyde solution for 30 min. The fixed cells were stained with AO (100 mg/mL, Sigma Aldrich, USA) for 30 min and with DAPI (Sigma Aldrich, USA) for 5 min. The cells were then washed three times with phosphate-buffered saline, and the slides were examined under a fluorescence microscope (Olympus, Tokyo, Japan). The results obtained were compared with this control group, which received no treatment.

### 2.6. Real Time-Polymerase Chain Reaction (RT-PCR) Analysis

To evaluate the effect of RUT and NaBu, individually and in combination, on the mRNA expression levels of *Bax*, *Bcl-2*, and *SOD* genes, RT-PCR analysis was conducted. PC-3 cells (4 × 10⁶ cells/well) were cultured in T25 flasks and treated with RUT (500 and 750 μM) and/or NaBu (1 and 2.5 mM) for 48 h. Additionally, a control group was established, cultured under the same conditions but without the addition of RUT, NaBu, or their combination. Following the incubation period, RNA isolation was performed using the Xtrazol solution (Hölzel, Cologne, Germany) according to the manufacturer’s protocol. RNA concentrations were quantified by measuring the absorbance at 260 nm using a spectrophotometer (Nanodrop, Thermo Fisher Scientific, USA). A total of 2 μg of RNA was reverse-transcribed into cDNA using the Tetro cDNA Synthesis Kit (Meridian Bioscience, Cincy, OH, USA). RT-PCR analysis was performed with a CFX Connect Real-Time PCR Detection System (Bio-Rad, Hercules, CA, USA) to assess the mRNA expression levels of *Bax*, *Bcl-2*, and *SOD*. *ACTB* (Beta-actin; Santa Cruz Biotechnology, Dallas, TX, USA) was used as the reference gene for the normalization of target gene expression. Each experiment was conducted in triplicate to ensure reproducibility.

### 2.7. Statistical Analysis

The statistical software GraphPad Prism V9.0 and SPSS 22.0 were used and *p* < 0.05 was considered statistically significant. A one-way ANOVA analysis with variance Tukey’s test was used for multiple comparisons. Web-based software https://www.qiagen.com/tr/shop/genes-andpathways/data-analysis-center-overviewpage/other-real-time-pcrprobes-orprimersdataanalysis-center/, (accessed on 20 May 2022) was used to determine the differences in the mRNA expression levels that vary depending on dose and time. Two-way repetitive measurements were performed with ANOVA analysis to determine the best combination concentrations. Additionally, the Calcusyn software V2.0 (Biosoft, Inc., Orlando, FL, USA) was used to determine the relationship between RUT and NaBu and the combination index (CI) values.

## 3. Results

### 3.1. The Cytotoxicity of RUT and NaBu Treatment in PC-3 Cells

**Figure 1:**
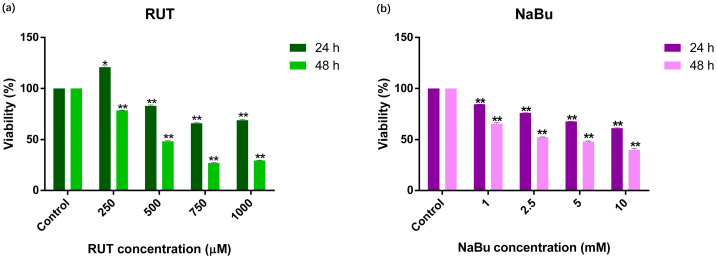
The viability rate of PC-3 prostate cancer cells after treatment with RUT or NaBu. (**a**) PC-3 cells were treated with RUT at concentrations of 250, 500, 750, or 1000 μM for 24 and 48 h. (**b**) PC-3 cells were treated with NaBu at concentrations of 1, 2.5, 5, or 10 mM for 24 and 48 h. Cell viability was determined using the WST-1 assay. Data are presented as the mean ± SD (n = 3) and are representative of experiments performed in triplicate. Statistical significance is indicated (* *p* < 0.05, **: Statistically significant, *p* ≤ 0.001).

To identify the most effective concentrations and exposure times for RUT and NaBu, a WST-1 assay was conducted (Figure 1). The results revealed that RUT exhibited a dose- and time-dependent cytotoxic effect on PC-3 cells, as shown in Figure 1a. Specifically, a treatment with higher concentrations of RUT (500, 750, and 1000 μM) reduced PC-3 cell viability by 48.47%, 26.92%, and 29.48%, respectively, after 48 h (Figure 1a, Table 1, *p* < 0.001). Similarly, NaBu treatment (1, 2.5, 5, and 10 mM) also decreased cell viability in a dose-dependent manner, with reductions of 65.58%, 52.34%, 48.15%, and 39.86%, respectively, after 48 h (Figure 1b, Table 1, *p* < 0.001). Based on these results, RUT concentrations of 500 and 750 μM, along with NaBu concentrations of 1, 2.5, 5, and 10 mM, were selected for combination treatment analysis over a 48 h period. 

### 3.2. The Effect of a Combined Treatment of RUT with NaBu on Cytotoxicity, Apoptosis, and Intracellular ROS Generation

**Figure 2:**
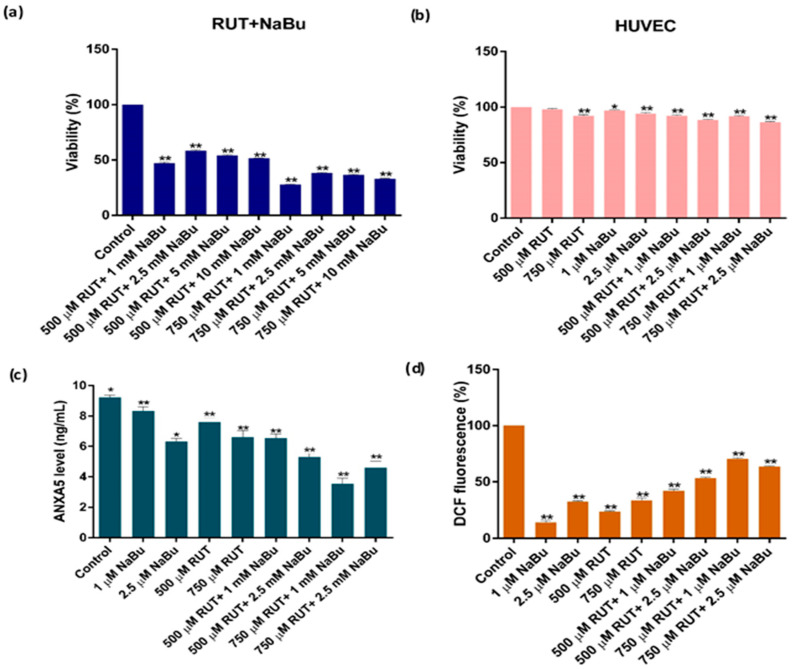
The effect of the combination treatment of RUT with NaBu on cell viability, apoptosis, and intracellular ROS generation. Cells were treated with RUT (500 and 750 μM) and/or NaBu (1, 2.5, 5, and 10 mM) for 48 h. (**a**) PC-3 and (**b**) HUVEC cell viability was assessed using the WST-1 assay. (**c**) Apoptosis was measured using the ANXA5 V ELISA assay. (**d**) Intracellular ROS generation was evaluated using the H2DCFDA assay, with fluorescence intensity indicating ROS levels. Data are presented as the mean ± SD (n = 3) and are representative of experiments performed in triplicate. Statistical significance is denoted as * *p* < 0.05 and ** *p* < 0.001.

To determine the most effective combined concentrations of RUT and NaBu, WST-1 analysis was performed (Figure 2). The combination of RUT and NaBu significantly reduced PC-3 cell viability compared to either treatment alone after 48 h (Table 2, *p* < 0.001). Specifically, the combinations of 500 μM RUT + 1 mM NaBu, 500 μM RUT + 2.5 mM NaBu, 750 μM RUT + 1 mM NaBu, and 750 μM RUT + 2.5 mM NaBu decreased PC-3 cell viability to 47.29%, 58.42%, 27.93%, and 38.33%, respectively (Table 2, *p* < 0.001), which were more effective than NaBu alone (65.52% and 52.34% for 1 and 2.5 mM NaBu, respectively) (Figure 2a). Notably, the RUT and NaBu combination treatments minimized NaBu’s cytotoxic effects on HUVEC cells, showing no significant toxicity (Table 2, Figure 2b, *p* < 0.001). Moreover, the combination treatments demonstrated significant synergism (CI < 1) at lower concentrations in PC-3 cells, making them substantially more effective than RUT or NaBu alone (Table 3 and Table 4). As a result, the combinations of 500 and 750 μM RUT with 1 and 2.5 mM NaBu for 48 h were selected for subsequent experiments in PC-3 cells.

The effects of RUT and NaBu combination treatments on apoptosis and intracellular ROS generation in PC-3 cells were assessed using ELISA and ROS analysis (Table 5, Figure 2c,d). The results revealed that the combination treatments significantly reduced free ANXA5 protein levels compared to individual treatments (Table 5, *p* < 0.001, Figure 2c). Similarly, RUT and NaBu combination treatments increased intracellular ROS levels more than either treatment alone (Table 5, *p* < 0.001, Figure 2d). Among the combinations, 750 μM RUT + 1 mM NaBu was the most effective in inducing both apoptosis and ROS production in PC-3 cells.

### 3.3. The Effect of Combined the Treatment of RUT with NaBu on Cell and Nucleus Morphology in PC-3 Cells

**Figure 3:**
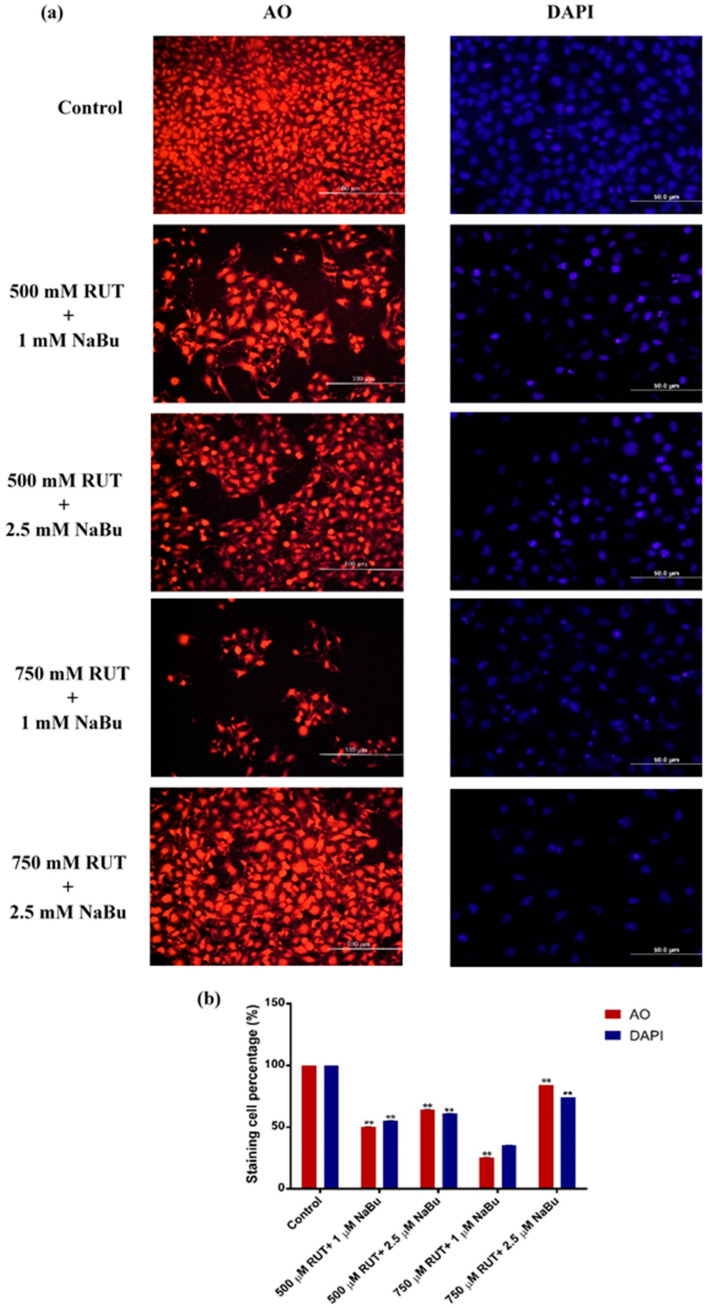
The effect of the combination treatment of RUT and NaBu on cell morphology and nuclear integrity. (**a**) PC-3 cells were treated with RUT (500 and 750 μM) in combination with NaBu (1 and 2.5 mM) for 48 h. Acridine orange (AO) staining was used to visualize cellular morphology, and 4′,6-diamidino-2-phenylindole (DAPI) staining was used to observe nuclear morphology. Fluorescence microscopy images were captured at 20× magnification for AO staining and 40× magnification for DAPI staining. Scale bar = 100 and 50 μm, respectively. (**b**) A quantitative analysis of AO and DAPI-positive cells is shown as a percentage. Data are presented as the mean ± SD (n = 3). ** *p* < 0.001 compared to control.

The combined effects of RUT and NaBu on cell and nuclear morphology in PC-3 cells were evaluated using AO and DAPI staining (Figure 3). Following treatment with RUT and NaBu combinations, apoptotic morphological changes, including a rounded cell shape, membrane blebbing, and cell shrinkage, were observed in PC-3 cells (Figure 3a). Among the treatments, the combination of 750 μM RUT + 1 mM NaBu was the most effective in inducing apoptotic cell morphology (Figure 3b, Table 6, *p* < 0.001). To further assess apoptotic cell death, the nuclear morphology of PC-3 cells was examined using DAPI staining (Figure 3a). The results revealed irregular nuclear shapes and nuclear condensation in PC-3 cells treated with RUT and NaBu combinations, particularly with the 750 μM RUT + 1 mM NaBu treatment (Figure 3b, Table 6, *p* < 0.001). These findings align with the ELISA and ROS analysis results, supporting the role of RUT and NaBu combinations in inducing apoptosis in PC-3 cells.

### 3.4. The Effect of the Combined Treatment of RUT with NaBu on mRNA Expression Levels

**Figure 4:**
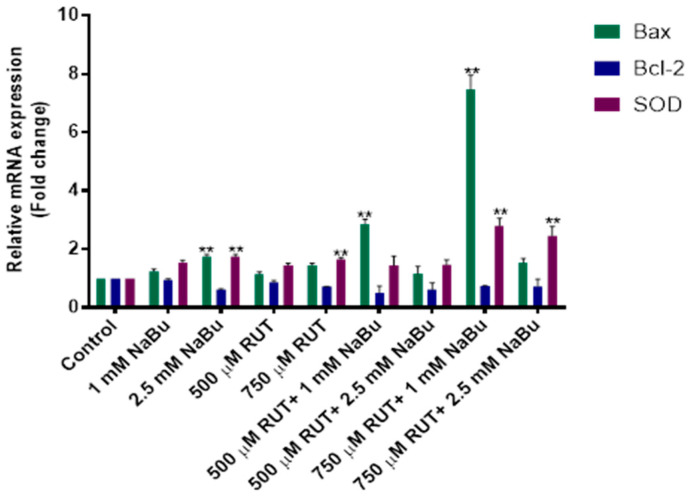
The effect of the combination treatment of RUT and NaBu on *Bax*, *Bcl-2*, and *SOD* mRNA expression in PC-3 cells. PC-3 cells were treated with RUT (500 μM and 750 μM) and NaBu (1 mM and 2.5 mM), either individually or in combination, for 48 h. The relative mRNA expression levels of *Bax*, *Bcl-2*, and *SOD* were quantified using RT-PCR and normalized to the control group. Data are presented as fold changes (mean ± SD). Statistical significance is denoted by ** *p* ≤ 0.05.

To evaluate the apoptotic potential of the RUT and NaBu combination treatment, the relative mRNA expression levels of *Bax* (pro-apoptotic), *Bcl-2* (anti-apoptotic), and *SOD* (antioxidant) were quantified using RT-PCR and normalized to the control group. (Figure 4). The combination treatment of 750 μM RUT + 1 mM NaBu for 48 h resulted in a significant increase in the relative expression levels of *Bax* and *SOD*, with 7.4- and 2.8-fold increases, respectively. Conversely, the mRNA expression level of *Bcl-2* was reduced by 0.7-fold (Figure 4, Table 7, *p* < 0.01). A significant upregulation of *Bax* mRNA expression was observed, particularly in the combination treatment of 750 μM RUT and 2.5 mM NaBu, suggesting a synergistic pro-apoptotic effect. No significant upregulation of *Bcl-2* was noted, while *SOD* showed moderate changes under some conditions. These findings indicate that the 750 μM RUT + 1 mM NaBu combination treatment exhibits a greater potency and synergistic cytotoxicity against metastatic prostate cancer cells.

## 4. Discussion

This study revealed that the combination therapy of RUT and NaBu had a significantly stronger inhibitory effect on PC-3 cells compared to their separate administrations, primarily by inducing apoptosis. Moreover, the combined treatment increased cellular ROS levels more effectively than either RUT or NaBu alone. The synergistic effect observed in this combination therapy not only enhanced its therapeutic efficacy but also reduced the systemic toxicity associated with NaBu. This highlights the potential of RUT and NaBu combination therapy as a potent and synergistic approach to cytotoxicity in mCRPC cells.

The use of natural plant extracts and flavonoids has demonstrated great potential in addressing various health challenges [10,11]. Among bioflavonoids, RUT is one of the most extensively studied, with significant contributions to inhibiting various types of cancers [12,16]. In this investigation, RUT was shown to dramatically reduce the viability of PC-3 cells, consistent with findings from Satari et al. [16], who reported the anti-proliferative effects of RUT on PC-3 cells. Although there is no prior research on the combined effects of RUT and NaBu, studies involving RUT in combination with other chemotherapeutic agents have shown highly effective results in various cancer cells [17,18]. In this study, the interaction of RUT and NaBu demonstrated a synergistic effect with a combination index (CI) as low as 0.28, which is even lower than the CI of 0.33 reported by Satari et al. [12] for the combination of RUT and 5-FU in PC-3 cells. In a recent study, we investigated the combined cytotoxic effects of sodium butyrate (NaBu) and the flavonoid quercetin on MCF-7 breast cancer cells and a synergistic interaction between NaBu and quercetin was observed, resulting in enhanced cytotoxicity compared to individual treatments [19]. These findings suggest that combining NaBu with quercetin could be a promising therapeutic strategy for targeting MCF-7 cells. Similar to our previous study, the results of this study also indicate that the combination of RUT and NaBu not only reduced NaBu’s systemic toxicity but also retained its therapeutic efficacy.

The synergistic interactions between flavonoids and various drugs are mediated by multiple molecular mechanisms, such as enzyme inhibition, the modulation of drug efflux pumps, induction of apoptosis, and anti-inflammatory as well as antioxidant activities [20,21,22,23]. These mechanisms underscore the therapeutic potential of flavonoid–drug combinations. For instance, certain flavonoid mixtures demonstrate significant synergistic effects on α-glucosidase inhibition, a key enzyme in carbohydrate metabolism, through the formation of hydrophobic interactions and hydrogen bonds with its active sites [20]. Additionally, taxifolin has been found to inhibit the overexpression of P-glycoprotein, a drug efflux protein, thereby preventing chemoresistance by blocking the action of rhodamine 123 and doxorubicin [21]. The co-administration of flavonoids with paclitaxel has also been shown to enhance the efficacy of chemotherapy by inducing apoptosis in cancer cells [22]. Furthermore, the combination of total saponins and flavonoids has displayed synergistic effects in reducing inflammation and myocardial cell apoptosis [23]. Although direct studies on the combined effects of NaBu and RUT are limited, research on similar compounds suggests potential synergistic mechanisms and the obtained data also suggested that NaBu and RUT synergistically increased apoptosis for the first time. However, further studies are required to clarify the specific interactions between NaBu and RUT, especially the downstream signaling cascades that control apoptosis.

Apoptosis is a crucial genetic process that ensures growth and cellular equilibrium. Cancer cells, however, evade apoptosis through mechanisms such as angiogenesis, uncontrolled proliferation, and the suppression of the intrinsic apoptotic pathway [24,25]. The primary function of apoptosis is to prevent cancer [26]. Cancer cells can inhibit apoptosis in various ways, such as blocking caspase activation or preventing the initiation of apoptosis. The overexpression of anti-apoptotic *Bcl-2* proteins and/or inactivation of pro-apoptotic proteins such as *Bax* or *Bak* are common mechanisms of evasion. While Bcl-2 is not classified as an oncogene, its altered expression contributes to malignancy development. An overexpression of Bcl-2 has been observed in more than 50% of cancer cases, regardless of subtype [27]. The findings of this study are consistent with those of Satari et al. [12], who reported that RUT, when combined with 5-FU, reduced Bcl-2 protein levels in prostate cancer cells. Similarly, Taylor et al. [27] found that NaBu, either alone or in combination with quercetin, significantly reduced cell growth or increased apoptosis by activating the pro-apoptotic *Bax* gene in human T986 glioblastoma cells. Additionally, a study investigating the effects of Curcuminoids and sodium butyrate, both individually and in combination, on three glioblastoma cell lines revealed that their combined treatment synergistically decreased glioblastoma cell viability [28]. This effect was achieved by inducing apoptosis, causing cell cycle arrest, and modulating ROS production and gene expression [28].

Antioxidant systems, comprising both enzymatic and non-enzymatic components, play a critical role in regulating ROS levels to mitigate oxidative stress. Cells adapt by activating antioxidant enzymes such as superoxide dismutase (SOD), which neutralizes ROS and minimizes damage [29,30]. During oxidative stress, these enzymes are often upregulated to counteract potential harm. RUT has been shown in several studies to reduce lipid peroxidation and alleviate oxidative stress [12,28]. Similarly, NaBu has been reported to exhibit genotoxic and anticancer effects, partly through its modulation of antioxidant enzymes [8]. In this study, the combination of RUT and NaBu significantly upregulated *SOD* expression compared to their separate administrations, suggesting that this combination effectively enhances the cellular capacity to combat oxidative damage by neutralizing free radicals.

## 5. Conclusions

NaBu is often administered as a combination therapy with other chemotherapeutic drugs to reduce the required therapeutic dose, thereby minimizing its toxic properties and clinical side effects. RUT, when administered alone, has demonstrated the ability to inhibit several aberrant signaling pathways associated with apoptosis, inflammation, autophagy, and angiogenesis, employing diverse mechanisms to impede cancer progression and metastasis. Our initial findings indicate that the combination therapy of RUT and NaBu holds considerable promise as an anticancer therapeutic approach and warrants further molecular investigation, particularly focusing on its effects on pro- and anti-apoptotic pathways. However, further research, including studies in animal models or other cell lines, is necessary to fully evaluate and optimize its potential efficacy in cancer treatment.

## Figures and Tables

**Table 1:** The statistical analysis results of PC-3 cell viability after treatment with RUT or NaBu. A two-way ANOVA analysis with Tukey’s multiple comparisons test was used for multiple comparisons. Statistical significance is denoted as ** *p* < 0.001.

RUT (µM)	Mean Difference	95% CI of Difference	Summary	Adjusted *p* Value
24 h				
Control vs. 250	−21.06	−23.14 to −18.98	**	<0.0001
Control vs. 500	16.97	14.89 to 19.05	**	<0.0001
Control vs. 750	34.02	31.95 to 36,10	**	<0.0001
Control vs. 1000	31.15	29.07 to 33.23	**	<0.0001
48 h				
Control vs. 250	21.59	19.51 to 23.67	**	<0.0001
Control vs. 500	51.53	49.45 to 53.61	**	<0.0001
Control vs. 750	73.07	70.99 to 75.15	**	<0.0001
Control vs. 1000	70.52	68.44 to 72.60	**	<0.0001
NaBu (mM)				
24 h				
Control vs. 1	15.35	13.83 to 16.87	**	<0.0001
Control vs. 2.5	23.96	22.44 to 25.48	**	<0.0001
Control vs. 5	32.34	30.82 to 33.86	**	<0.0001
Control vs. 10	38.87	37.35 to 40.39	**	<0.0001
48 h				
Control vs. 1	34.42	32.90 to 35.94	**	<0.0001
Control vs. 2.5	47.66	46.13 to 49.18	**	<0.0001
Control vs. 5	51.84	50.32 to 53.37	**	<0.0001
Control vs. 10	60.14	58.62 to 61.66	**	<0.0001

CI: confidence intervals.

**Table 2:** The statistical analysis results of PC-3 and HUVEC cell viability after combination treatment with RUT and NaBu. A two-way ANOVA analysis with Dunnett’s multiple comparisons test was used for multiple comparisons. Statistical significance is denoted as * *p* < 0.05 and ** *p* < 0.001.

RUT (µM) + NaBu (mM)	Mean Difference	95% CI of Difference	Summary	Adjusted *p* Value
PC-3				
Control vs. RUT 500 + NaBu 1	52.71	51.05 to 54.36	**	<0.0001
Control vs. RUT 500 + NaBu 2.5	41.57	39.92 to 43.22	**	<0.0001
Control vs. RUT 500 + NaBu 5	45.76	44.11 to 47.42	**	<0.0001
Control vs. RUT 500 + NaBu 10	48.41	46.76 to 50.06	**	<0.0001
Control vs. RUT 750 + NaBu 1	72.06	70.41 to 73.72	**	<0.0001
Control vs. RUT 750 + NaBu 2.5	61.67	60.01 to 63.32	**	<0.0001
Control vs. RUT 750 + NaBu 5	63.47	61.82 to 65.13	**	<0.0001
Control vs. RUT 750 + NaBu 10	66.97	65.32 to 68.62	**	<0.0001
HUVEC				
Control vs. RUT 500	2.102	−0.1289 to 4.333	ns	0.0699
Control vs. RUT 750	7.892	5.662 to 10.12	**	<0.0001
Control vs. NaBu 1	2.965	0.7339 to 5.196	*	0.0066
Control vs. NaBu 2.5	5.916	3.685 to 8.147	**	<0.0001
Control vs. RUT 500 + NaBu 1	7.809	5.578 to 10.04	**	<0.0001
Control vs. RUT 500 + NaBu 2.5	11.61	9.384 to 13.85	**	<0.0001
Control vs. RUT 750 + NaBu 1	8.273	6.042 to 10.50	**	<0.0001
Control vs. RUT 750 + NaBu 2.5	13.65	11.42 to 15.88	**	<0.0001

CI: confidence intervals, ns: no significance.

**Table 3:** The RUT and NaBu interaction in PC-3 cells was determined by CI values for 24 h.

RUT	500 µM	750 µM
CI at 1 mM Nabu	0.39	0.28
CI at 2.5 mM Nabu	0.74	0.46
CI at 5 mM Nabu	0.82	0.51
CI at 10 mM Nabu	1.13	0.59

CI: Combination index, CI < 1 Synergism; CI = 1 Additive Effect; CI > 1 Antagonism.

**Table 4:** The combined treatment of RUT and NaBu was analyzed by dose reduction index (DRI) values for 24 h in PC-3 cells.

RUT	500 µM		750 µM	
	NaBu	RUT	NaBu	RUT
DRI at 1 mM Nabu	16.35	3.01	50.12	3.70
DRI at 2.5 mM Nabu	3.55	2.15	10.83	2.64
DRI at 5 mM Nabu	2.35	2.51	6.08	2.82
DRI at 10 mM Nabu	1.31	2.67	3.64	3.11

DRI: Dose reduction index; DRI < 1: not favorable dose reduction; DRI = 1: no-dose reduction; DRI > 1: favorable dose reduction.

**Table 5:** The statistical analysis results of ANXA5 V ELISA and ROS generation in PC-3 cells after the combination treatment of RUT with NaBu. A two-way ANOVA analysis with Tukey’s multiple comparisons test was used for multiple comparisons. Statistical significance is denoted as * *p* < 0.05 and ** *p* < 0.001.

	Mean Difference	95% CI of Difference	Summary	Adjusted *p* Value
ANXA5 ELISA				
Control vs. 1 mM NaBu	0.8962	0.002110 to 1.790	*	0.0493
Control vs. 2.5 mM NaBu	2.914	2.020 to 3.808	**	<0.0001
Control vs. 500 mM RUT	1.628	0.7340 to 2.522	*	0.0003
Control vs. 750 mM RUT	2.619	1.725 to 3.513	**	<0.0001
Control vs. RUT 500 + NaBu 1	2.696	1.802 to 3.590	**	<0.0001
Control vs. RUT 500 + NaBu 2.5	3.914	3.020 to 4.808	**	<0.0001
Control vs. RUT 750 + NaBu 1	5.678	4.784 to 6.572	**	<0.0001
Control vs. RUT 750 + NaBu 2.5	4.619	3.725 to 5.513	**	<0.0001
ROS				
Control vs. 1 mM NaBu	86.00	82.13 to 89.87	**	<0.0001
Control vs. 2.5 mM NaBu	67.50	63.63 to 71.37	**	<0.0001
Control vs. 500 mM RUT	76.50	72.63 to 80.37	**	<0.0001
Control vs. 750 mM RUT	66.50	62.63 to 70.37	**	<0.0001
Control vs. RUT 500 + NaBu 1	58.00	54.13 to 61.87	**	<0.0001
Control vs. RUT 500 + NaBu 2.5	46.50	42.63 to 50.37	**	<0.0001
Control vs. RUT 750 + NaBu 1	29.50	25.63 to 33.37	**	<0.0001
Control vs. RUT 750 + NaBu 2.5	36.50	32.63 to 40.37	**	<0.0001

CI: confidence intervals, ns: no significance.

**Table 6:** The statistical analysis results of AO and DAPI staining in PC-3 cells after the combination treatment of RUT with NaBu. A two-way ANOVA analysis with Dunnett’s multiple comparisons test was used for multiple comparisons. Statistical significance is denoted as ** *p* < 0.001.

RUT (µM) + NaBu (mM)	Mean Difference	95% CI of Difference	Summary	Adjusted *p* Value
AO staining cells				
Control vs. RUT 500 + NaBu 1	49.80	49.02 to 50.58	**	<0.0001
Control vs. RUT 500 + NaBu 2.5	35.80	35.02 to 36.58	**	<0.0001
Control vs. RUT 750 + NaBu 1	74.70	73.92 to 75.48	**	<0.0001
Control vs. RUT 750 + NaBu 2.5	15.90	15.12 to 16.68	**	<0.0001
DAPI staining cells				
Control vs. RUT 500 + NaBu 1	44.80	44.02 to 45.58	**	<0.0001
Control vs. RUT 500 + NaBu 2.5	38.80	38.02 to 39.58	**	<0.0001
Control vs. RUT 750 + NaBu 1	64.70	63.92 to 65.48	**	<0.0001
Control vs. RUT 750 + NaBu 2.5	25.90	25.12 to 26.68	**	<0.0001

CI: confidence intervals.

**Table 7:** The statistical analysis results of a RT-PCR analysis in PC-3 cells after combination treatment of RUT with NaBu. A two-way ANOVA analysis with Tukey’s multiple comparisons test was used for multiple comparisons. Statistical significance is denoted as * *p* < 0.05 and ** *p* < 0.001.

	Mean Difference	95% CI of Difference	Summary	Adjusted *p* Value
Bax				
Control vs. 1 mM NaBu	−0.2500	−0.7507 to 0.2507	ns	0.6124
Control vs. 2.5 mM NaBu	−0.7500	−1.251 to −0.2493	*	0.0016
Control vs. 500 mM RUT	−0.1500	−0.6507 to 0.3507	ns	0.9431
Control vs. 750 mM RUT	−0.4500	−0.9507 to 0.05066	ns	0.0925
Control vs. RUT 500 + NaBu 1	−1.870	−2.371 to −1.369	**	<0.0001
Control vs. RUT 500 + NaBu 2.5	−0.1650	−0.6657 to 0.3357	ns	0.9109
Control vs. RUT 750 + NaBu 1	−6.480	−6.981 to −5.979	**	<0.0001
Control vs. RUT 750 + NaBu 2.5	−0.5450	−1.046 to −0.04434	*	0.0283
Bcl-2				
Control vs. 1 mM NaBu	0.05000	−0.4507 to 0.5507	ns	0.9996
Control vs. 2.5 mM NaBu	0.3750	−0.1257 to 0.8757	ns	0.2108
Control vs. 500 mM RUT	0.1300	−0.3707 to 0.6307	ns	0.9730
Control vs. 750 mM RUT	0.2750	−0.2257 to 0.7757	ns	0.5142
Control vs. RUT 500 + NaBu 1	0.4950	−0.005658 to 0.9957	ns	0.0537
Control vs. RUT 500 + NaBu 2.5	0.3850	−0.1157 to 0.8857	ns	0.1901
Control vs. RUT 750 + NaBu 1	0.2600	−0.2407 to 0.7607	ns	0.5725
Control vs. RUT 750 + NaBu 2.5	0.2700	−0.2307 to 0.7707	ns	0.5334
SOD				
Control vs. 1 mM NaBu	−0.5500	−1.051 to −0.04934	*	0.0265
Control vs. 2.5 mM NaBu	−0.7500	−1.251 to −0.2493	*	0.0016
Control vs. 500 mM RUT	−0.4500	−0.9507 to 0.05066	ns	0.0925
Control vs. 750 mM RUT	−0.6500	−1.151 to −0.1493	*	0.0067
Control vs. RUT 500 + NaBu 1	−0.4450	−0.9457 to 0.05566	ns	0.0980
Control vs. RUT 500 + NaBu 2.5	−0.4700	−0.9707 to 0.03066	ns	0.0729
Control vs. RUT 750 + NaBu 1	−1.795	−2.296 to −1.294	**	<0.0001
Control vs. RUT 750 + NaBu 2.5	−1.460	−1.961 to −0.9593	**	<0.0001

CI: confidence intervals; ns: no significance.

## Data Availability

Data are contained within the article.

## Abbreviations

The following abbreviations are used in this manuscript:

RUT	Rutin
NaBu	Sodium Butyrate
PCa	Prostate Cancer
ROS	Reactive oxygen species
mCRPC	Metastatic castration-resistant prostate cancer
AR	Androgen receptors
